# Rapidly growing glandular papilloma associated with mucus production: a case report

**DOI:** 10.1186/1477-7819-12-160

**Published:** 2014-05-22

**Authors:** Shigeki Suzuki, Taichiro Goto, Katsura Emoto, Yuichiro Hayashi

**Affiliations:** 1 Division of General Thoracic Surgery, Department of Surgery, School of Medicine, Keio University, 35 Shinanomachi, Shinjuku-ku Tokyo 160-8582, Japan; 2 Department of Pathology, School of Medicine, Keio University, 35 Shinanomachi, Shinjuku-ku Tokyo 160-8582, Japan

## Abstract

**Background:**

Pulmonary glandular papillomas are rare neoplasms, and their very slow or absent growth over time generally facilitates establishing the diagnosis.

**Case presentation:**

In an 84-year-old woman who underwent surgery for sigmoid colon cancer, a growing solitary pulmonary nodule was identified on postoperative follow-up computed tomography. A computer tomography-guided needle biopsy was performed under suspicion that the nodule was malignant. The histopathological findings suggested a glandular papilloma. Right basilar segmentectomy was carried out, and the lesion was completely resected. Postoperative histopathological examination revealed a benign glandular papilloma accompanied by mucus retention in the surrounding alveolar region.

**Conclusions:**

A malignant neoplasm is usually suspected when a pulmonary tumor shows rapid growth. However, glandular papillomas associated with mucus retention also tend to grow in some cases, and should be included in the differential diagnosis.

## Background

Solitary pulmonary papillomas are rare neoplasms usually arising from the bronchial surface epithelium and forming endobronchial tumors [1-3]. Most cases have squamous cell papillomas, but papillomas lined by a ciliated or nonciliated columnar epithelium are termed glandular papillomas [1,4]. The latter type is reported infrequently [5]. There are very few reports in the literature describing solitary papillomas arising in peripheral bronchioles [2,6,7]. The differential diagnosis of glandular papilloma usually includes bronchioloalveolar or papillary-type adenocarcinomas, but the possibility of a glandular papilloma associated with mucus retention is seldom considered. Herein, we present a surgical case with a solitary glandular papilloma located in a bronchiolar region, which grew rapidly and thus required differential diagnosis from papillary-type adenocarcinoma.

## Case presentation

**Figure 1:**
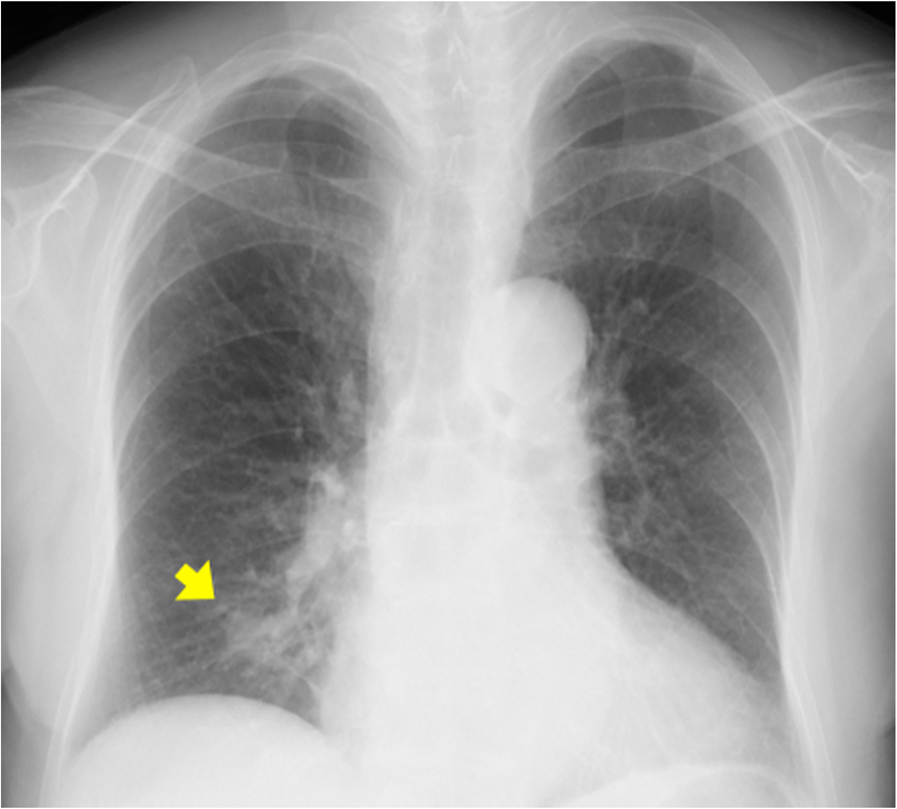
**Chest X-ray shows an abnormal shadow in the right lower lung field.** The lesion is indicated by the arrow.

**Figure 2:**
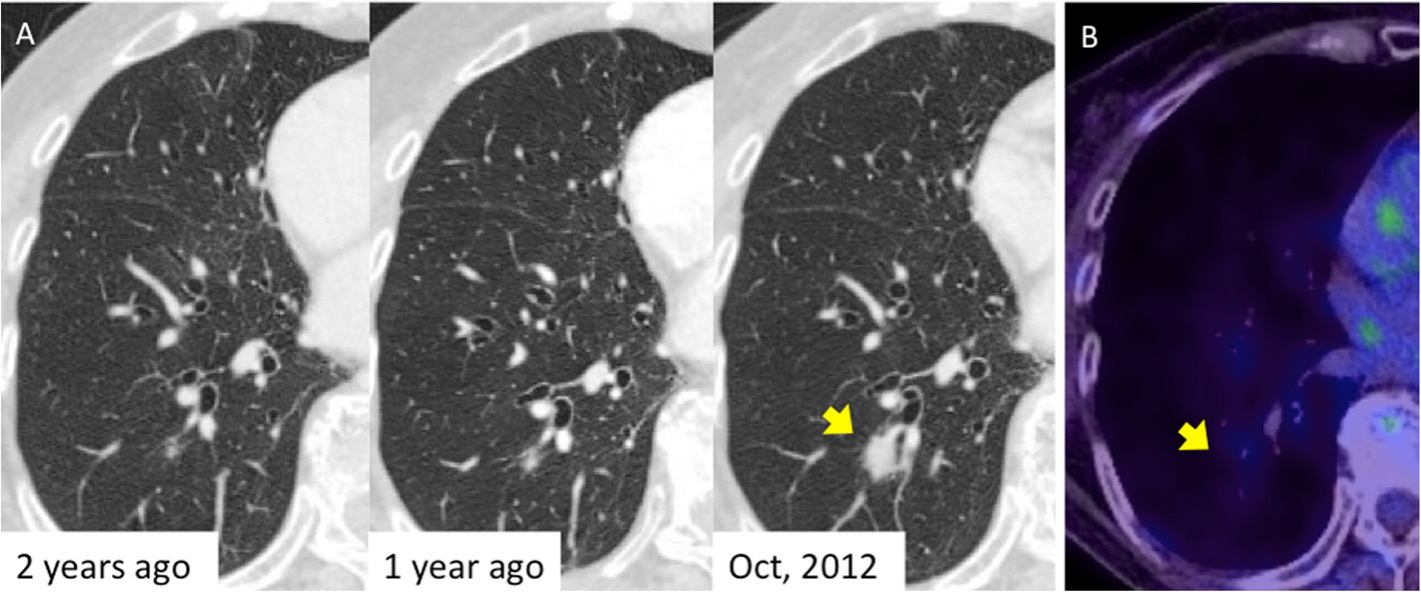
**Radiological findings of the nodule. (A)** Chest computed tomography (CT) shows an irregularly shaped nodule in the right lower lobe. A tiny nodule at the same site had been found two years earlier. The nodule had since shown a clear tendency to increase in size. The arrow points to the lesion. **(B)** Positron emission tomography (PET)-CT shows slight accumulation of fluorodeoxyglucose in the nodule (SUVmax = 1.5). The lesion is indicated by the arrow.

**Figure 3:**
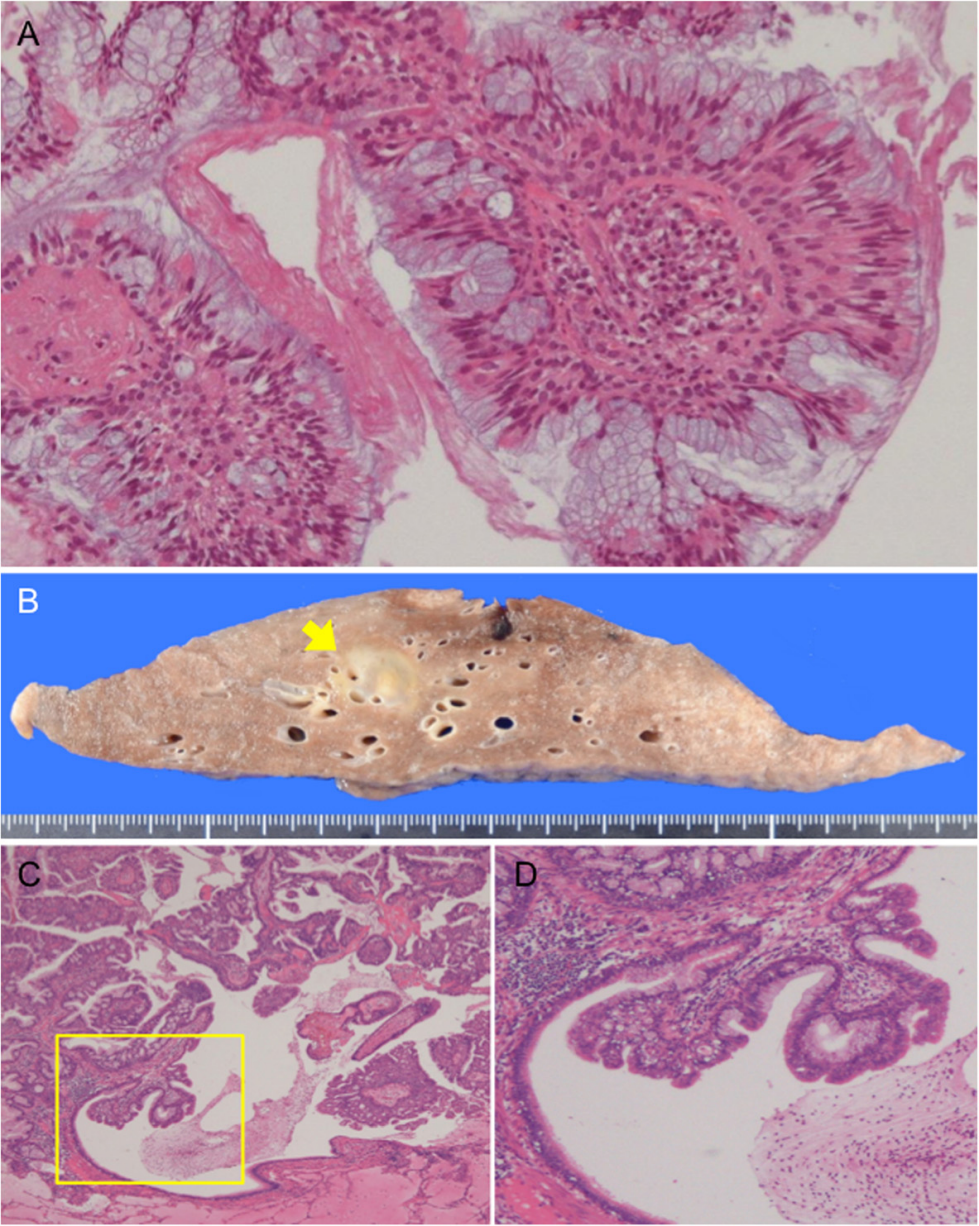
**Pathological findings. (A)** Computed tomography-guided needle biopsy reveals the tumor to have papillary stromal cores lined by pseudostratified ciliated or nonciliated columnar cells admixed with mucin-filled cells. The tumor cells show minimal atypia. **(B)** Cut surface of the resected specimen shows a whitish mucinous portion and a yellowish tumor cell portion in the center. The lesion is indicated by the arrow. **(C)** Histopathological image of the resected specimen shows papillomatous fronds lined by columnar epithelium and their extension toward the bronchiolar lumen. Surrounding alveoli are filled with mucus. **(D)** High magnification of the yellow box in **C** shows thick fibrovascular cores surrounded by goblet cells. The endobronchiolar surface of the tumor is covered mainly by ciliated epithelium with minimal atypia.

An 84-year-old woman was found to have an abnormal shadow on a chest X-ray during postoperative follow-up for colon cancer (Figure 1), and was referred to our department in December of 2012. She was a never-smoker. Chest computed tomography (CT) showed a 17-mm irregularly shaped tumor in the right segment 9 (Figure 2A). Positron emission tomography (PET) showed neither abnormal fluorodeoxyglucose uptake in the tumor nor evidence of metastatic foci elsewhere in the body (Figure 2B). Blood chemistry data were unremarkable, and all tumor markers were within normal limits. She was otherwise in good health.A review of previous chest CT images demonstrated that there had been a nodular lesion at the same site two years before, which had grown relatively rapidly during the year prior to the current presentation (Figure 2A). The nodule measured 3 mm, 5 mm and 17 mm in maximum diameter on chest CT, two years ago, one year ago and in October 2012, respectively. Considering its clear tendency to increase in size, a primary lung cancer was suspected. CT-guided needle biopsy revealed that, histologically, the tumor had central fibrovascular cores, and there was hyperplasia of goblet cells and columnar ciliated or nonciliated epithelium (Figure 3A). Because no structural atypia or cytological atypia corresponding to adenocarcinoma was detected, a glandular papilloma was initially suspected. Tumor cells were thyroid transcription factor-1 positive, cytokeratin-7 positive, and cytokeratin-20 negative on immunostaining, indicating metastasis of colon cancer to be unlikely. This case was judged to be an appropriate candidate for surgery, as it is known that the biopsy specimens did not allow the possibility of malignancy to be definitely ruled out and as the tumor, even if benign, had growth toward the hilar region. Therefore, a right basilar segmentectomy was performed.

Macroscopically, a whitish mucous nodule measuring 17 mm in diameter with a yellowish endobronchiolar tumor approximately 5 mm in diameter in the center was observed (Figure 3B). Histopathologically, the tumor consisted of fibrovascular papillary cores lined by benign glandular epithelium. Consistent with the biopsy findings, there was no atypia corresponding to adenocarcinoma; glandular papilloma was thus diagnosed (Figure 3C-D). There was abundant mucus retention within the surrounding alveolar region. Mitoses and necrosis were absent, and the bronchial margins were free of tumor cells. *In situ* hybridization for human papilloma virus DNA was not performed in this case because there had been no reports of malignant transformation of glandular papillomas so far.

The patient had an uneventful postoperative course. At present, 12 months after lung surgery, she remains recurrence free.

## Discussion and conclusions

Papillomas of the lung have two clinical presentations: multiple and solitary [3,4]. Solitary respiratory papillomas are the rarer type, accounting for 0.38% of all lung tumors, and approximately 7% of all benign epithelial and mesenchymal lung tumors [8]. Multiple papillomas most often occur in children and young adults, while solitary respiratory papillomas usually affect middle-aged adults [4,7,9]. Solitary respiratory papillomas are histologically divided into three categories, that is, squamous cell, glandular, and mixed types [2,9]. Squamous cell papillomas are the most common type. Glandular papillomas are apparently rare with only 20 cases having been reported in the English literature since the first case report of bronchial glandular papilloma in 1954 by Ashmore [5,10].

Glandular papillomas occur predominantly in the central tracheobronchial tree [7,11]. They arise from the mucosal surface, and range in size from 0.7 to 2.5 cm [2]. At bronchoscopy, they appear as endobronchial masses, which cause narrowing of the airway lumen, to approximately 40% to 60% of its normal diameter [3,5]. On CT or chest X-ray, these lesions have findings of atelectasis, air trapping, postobstructive infections, and bronchiectasis. Glandular papillomas may produce obstructive symptoms, and the main symptom is reportedly coughing [5]. In some cases, solitary respiratory papillomas go undetected for years, being detected only by chance, as our presented patient [5]. It is expected that such incidental detection of solitary respiratory papillomas will increase as health screening becomes more common [6].

It might be difficult to distinguish a glandular papilloma from active inflammation, granulomatous disease, carcinoid, and lung cancer based only on imaging findings [11]. Bronchoscopic biopsy or CT-guided needle biopsy is necessary to confirm the diagnosis. In addition, the possibility of a malignant lesion often cannot entirely be ruled out before surgery, and a definite diagnosis usually is only obtained by pathological examination of the resected tumor. Pathologically, papillomas consist of fibrovascular papillary cores lined by benign epithelium. Papillomas covered entirely by mucous, simple columnar or ciliated cells are termed glandular papillomas [2]. For differential diagnosis, it is noteworthy that endobronchiolar papillomatous fronds are consistently seen and spread along alveolar walls is limited in adjacent alveoli. Glandular papillomas seldom show necrosis, and the presence of ciliated cells is considered to be an important finding for ruling out peripheral well-differentiated adenocarcinoma [2]. We can reasonably speculate that, in our case, a glandular papilloma arose in a peripheral bronchiole, and mucus retention in the surrounding alveolar region occurred along with microinvasion of tumor cells into the lung, leading to the rapid increase in nodule size. Glandular papillomas of this type have not previously been reported. We believe that the very rare pathological features of our present case may shed light on the pathology underlying glandular papilloma onset and growth.

Although there are occasional recurrent cases, most glandular papillomas are cured by excision only [12]. There have been no reports of malignant transformation of glandular papillomas, in contrast with squamous cell papillomas, which have malignant potential [5]. In the absence of demonstrable malignant potential, conservative management of glandular papillomas seems justifiable, in contrast to surgical resection that is favored for squamous cell papillomas [1]. The best and most appropriate treatment for glandular papilloma has not as yet been established, and whether to perform surgery must be decided on an individual basis, according to clinical manifestations and the location, morphology, and time course of the tumor. In our case, CT-guided needle biopsy provided findings suggestive of glandular papilloma. However, given the possibility of malignancy and the risk of atelectasis or obstructive pneumonia development resulting from rapid growth of the tumor, surgical resection was deemed to be necessary. Further accumulation of glandular papilloma cases is desirable to better understand the pathological condition underlying this disease and to establish an appropriate treatment algorithm.

## Consent

Written informed consent was obtained from the patient for the publication of this case presentation and any accompanying images. A copy of the written consent is available for review by the Editor-in-Chief of this journal.

## Abbreviations

CT: computed tomography; PET: positron emission tomography.

## Competing interests

The authors declare that they have no competing interests.

## Authors’ contributions

SS and TG wrote the manuscript. SS and TG performed the surgery. KE and YH carried out the pathological examination. TG was involved in the final editing. All authors approved the final manuscript.
